# Effects of Anterior Borderzone Angle Grading on Predicting the 90-Day Prognosis After Recanalization of Acute Middle Cerebral Artery Occlusion

**DOI:** 10.3389/fneur.2021.700732

**Published:** 2021-08-26

**Authors:** Ying Chen, Quanlong Hong, Junpeng Liu, Zhen Zheng, Yingchao He, Shuheng Chen, Canxiong Wang, Mengjuan Cai, Qiong Cheng, Yinzhou Wang, Yongkun Li

**Affiliations:** ^1^Department of Neurology, Fujian Provincial Hospital, Shengli Clinical College of Fujian Medical University, Fuzhou, China; ^2^Department of Neurology, The First Hospital of Quanzhou Affiliated to Fujian Medical University, Quanzhou, China; ^3^Fujian Academy of Medical Sciences, Fujian Key Laboratory of Medical Measurement, Fuzhou, China; ^4^Department of Neurology, Xiamen Key Laboratory of Brain Center, The First Affiliated Hospital of Xiamen University, Xiamen, China

**Keywords:** anterior borderzone angle grading, collateral circulation, endovascular treatment, acute ischemic stroke, prognosis

## Abstract

**Objective:** This work explores collateral circulation metrics, such as the anterior borderzone angle grading (ABZA-grading), as a predictor of the prognosis in patients with acute middle cerebral artery occlusion (MCAO) following endovascular treatment (EVT).

**Methods:** Clinical data from 108 patients with acute MCAO, treated by EVT, were retrospectively analyzed. In patients with MCAO, ABZA is the angle between the median line of the sagittal sinus and the borderzone of the pial arterioles of ACA and MCA, and the ABZA/23.0° was rounded to obtain the corresponding collateral circulation score (ABZA-grading). In parallel, the primary outcome was defined as the 90-day clinical outcome by modified ranking scale score (mRS). Univariate analysis and logistic regression were used to analyze the independent predictors of the 90-day clinical outcome (mRS). Receiver operating characteristic curve (ROC) analysis was used to judge the predictive value of ABZA.

**Results:** Univariate analysis and logistic regression analysis showed that ABZA-grading > 2 and age were independent predictors of the 90-day clinical outcome after EVT in patients with acute MCAO. The ROC analysis showed that ABZA alone could predict a favorable 90-day clinical outcome with an area under the curve (AUC) of 0.868. Using an ABZA of >57.8° (the corresponding ABZA-grading of >2) as the cut-off value, the predictive sensitivity and specificity were 75.7 and 88.7%, respectively. Contingency table analysis showed a statistical difference in mRS score between ABZA-grading subgroups, and ABZA-grading between stroke caused by large artery atherosclerosis (LAA) and cardiogenic embolism (CE).

**Conclusion:** The ABZA-grading is an easy and objective assessment of collateral circulation that is independently associated with short-time clinical outcome after EVT in patients with acute MCAO. Therefore, it may guide selection of patients with acute ischemic stroke (AIS) suitable for EVT. The ABZA-grading of collateral circulation can be a supplemental metric to help differentiate stroke by LAA and CE.

## Introduction

Numerous studies have shown that good collateral circulation is essential to maintain ischemic penumbra, and is associated with smaller infarct cores and improved clinical outcomes after intravenous and intrarterial thrombolysis. Contrastingly, poor collateralization increases mortality in patients with large vessel occlusion acute ischemic stroke (LVO-AIS) (1–4). Preserving ischemic penumbra, where collateral circulation plays an important role, is key to successful treatment. Therefore, it becomes important to evaluate collateral circulation before endovascular therapy (EVT).

Angiography is considered the gold standard for the assessment of collateral blood flow, providing complete and reliable information on the circle of willis and the leptomeningeal collateral circulation. At present, the scoring methods based on digital subtraction angiography (DSA) for assessing collateral state include: the American Society of Interventional and Therapeutic Neuroradiology/Society of Interventional Radiology (ASITN/SIR) and the Capillary Index Score (CIS). Of these two, ASITN/SIR-grading is most widely used because of its simplicity and operability. However, most of the collateral circulation scoring methods are subjective, and some studies show that the reproducibility between observers and repeatability by the same observer are weak (5). The middle cerebral artery (MCA) is the most frequently involved site of LVO-AIS, while the anterior cerebral artery (ACA) is the main compensatory source of pial collateral in MCA stenosis and occlusion. Our previous study introduced a new quantitative parameter of anterior borderzone angle (ABZA), to study changes of ACA-MCA boundary area; and exposed its relationship to hemodynamic changes during MCA stenosis and occlusion. Together these could be used as a quantitative index to evaluate the compensation of ACA pial collateral (6). Therefore, we tested the predictive value of this measurement ABZA (ABZA-grading), on the prognosis of patients with acute middle cerebral artery occlusion (MCAO) treated by EVT.

## Materials and Methods

**Figure 1:**
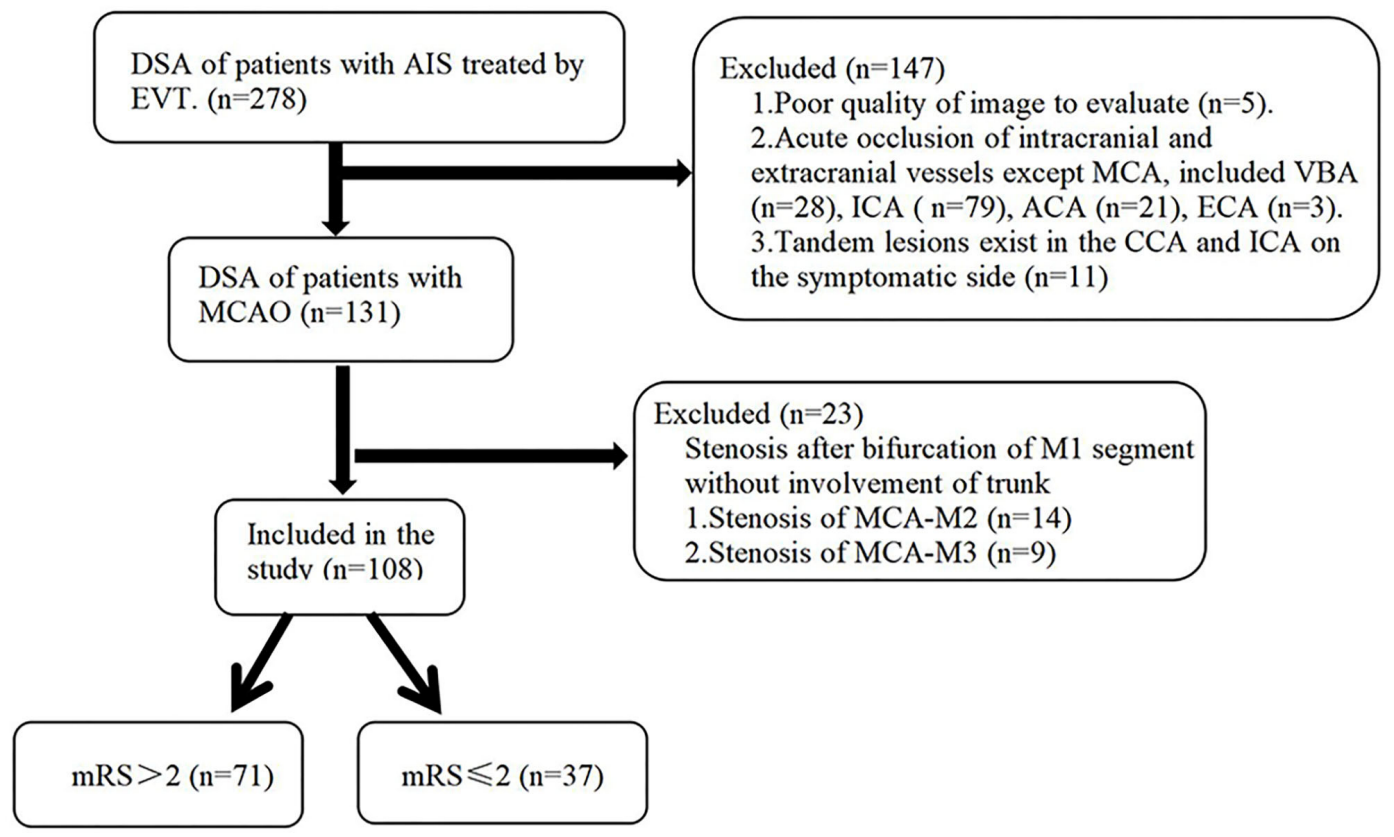
Block diagram of enrollment into this study. MCAO, middle cerebral artery occlusion; ABZA, anterior borderzone angle grading; mRS, modified Rankin score; PCA, posterior cerebral artery; ACA, anterior cerebral artery; CCA, common carotid artery; ECA, external carotid artery.

Data were collected from 69 patients attending the Department of Neurology at Fujian Provincial Hospital from June 2016 to November 2020 and 39 patients attending the First Hospital of Quanzhou City from April 2019 to November 2020, a total of 108 AIS patients from both hospitals receiving EVT who met the inclusion criteria. The Block diagram of enrollment was showed in Figure 1. Inclusion criteria were as follows: (1) All patients met the diagnostic criteria for AIS established by the American Heart Association/American Stroke Association (AHA/ASA) in 2018; (2) Age ≥ 18 years; (3) Treatment started within 6 h after symptoms onset; (4) M1 segment occlusion of the MCA was confirmed by computed tomography angiography (CTA) or DSA, and there was no forward blood flow; (5) Pre-onset mRS ≤ 2 points; NIHSS score ≥ 6 points on admission; (6) Early CT ischemic changes of Albert Stroke Program early CT score (ASPECTS) ≥ 6 or infarct volume <1/3 MCA blood supply area; (7) No contraindications to EVT and signed informed consent for EVT; (8) Patients whose symptoms did not resolve after intravenous thrombolysis and who met the above criteria could also be enrolled.

Exclusion criteria included: (1) Patients with severe stenosis or occlusion of other intracranial and extracranial vessels, including in the contralateral internal carotid or MCA or external carotid, anterior cerebral or posterior cerebral artery; (2) Tandem lesions exist in the common carotid artery and internal carotid artery on the symptomatic side; (3) Stenosis after bifurcation of M1 segment without involvement of trunk; (4) The image quality does not meet the analysis requirements; (5) All patients with the contraindications of EVT.

This study was approved by the Ethics Committee of Fujian Provincial Hospital and Quanzhou first Hospital and was conducted with the informed consent of patients or their legal representatives. Patients' information was analyzed retrospectively, including demographic characteristics (age, sex), stroke risk factors (hypertension, diabetes, hyperlipidemia, smoking, stroke, coronary heart disease), baseline clinical features (NIHSS score, systolic blood pressure, blood glucose, TOAST etiological classification), and imaging characteristics (baseline ASPECTS, collateral circulation score). The definition of TOAST etiological classification is based on the previous published study (7). Patients with large artery atherosclerosis (LAA) will meet the flowing criteria: (1) clinical and brain imaging findings of either significant (>50%) stenosis or occlusion of a major brain artery or branch cortical artery, presumably due to atherosclerosis. (2) Clinical findings include those of cerebral cortical impairment or brain stem or cerebellar dysfunction. (3) Cortical or cerebellar lesions and brain stem or subcortical hemispheric infarcts > 1.5 cm in diameter on CT or MRI. (4) Supportive evidence by duplex imaging or arteriography of a stenosis of >50% of an appropriate intracranial or extracranial artery is needed. (5) Excluding potential sources of cardiogenic embolism. And the patients with cardioembolism (CE) will meet the following criteria: (1) patients with at least one cardiac source of emboli. (2) Evidence of a previous TIA or stroke in more than one vascular territory or systemic embolism. (3) Eliminating the potential LAA sources of thrombosis or embolism. (4) A stroke in a patient with a medium-risk cardiac source of embolism and no other cause of stroke is classified as a possible cardioembolic stroke.

### Treatment Method

EVT was routinely performed according to the guidelines for EVT of acute ischemic cerebrovascular disease (8), and alteplase was given within the therapeutic time window when patients meet the criteria for intravenous thrombolysis. If there was a suspicion of excess embolus load, possible inefficacy of intravenous thrombolytic therapy or contraindication of intravenous thrombolytic therapy, the Neurointerventional Specialist could directly administer EVT with the informed consent of the patient.

### Surgical Method

Patient was placed in supine position, and punctured 1.5 cm under the pulsation of the right inguinal femoral artery. Anesthesia was adjusted to patients cooperation; local anesthesia for cooperative patients, and general anesthesia for those who could not cooperate. Cerebral angiography was performed first, the MCAO confirmed and then thrombectomy was performed according to general medical procedures. Thrombectomy can be was performed by simple aspiration, simple stent, or stent combined with aspiration treatment. Stent (4 × 20 mm or 6 × 30 mm Solitair FR stent, EV3, CA, USA), was selected according to vessel diameter and thrombus length. When necessary, Tirofiban was slowly injected when embolus could not be removed during embolectomy, or residual embolus or thrombus formation was observed during stent removal. If embolectomy was followed by re-occlusion, remedial treatment was taken, such as balloon dilation, intra-arterial thrombolysis or stent implantation.

### The Measurement of ABZA

**Figure 2:**
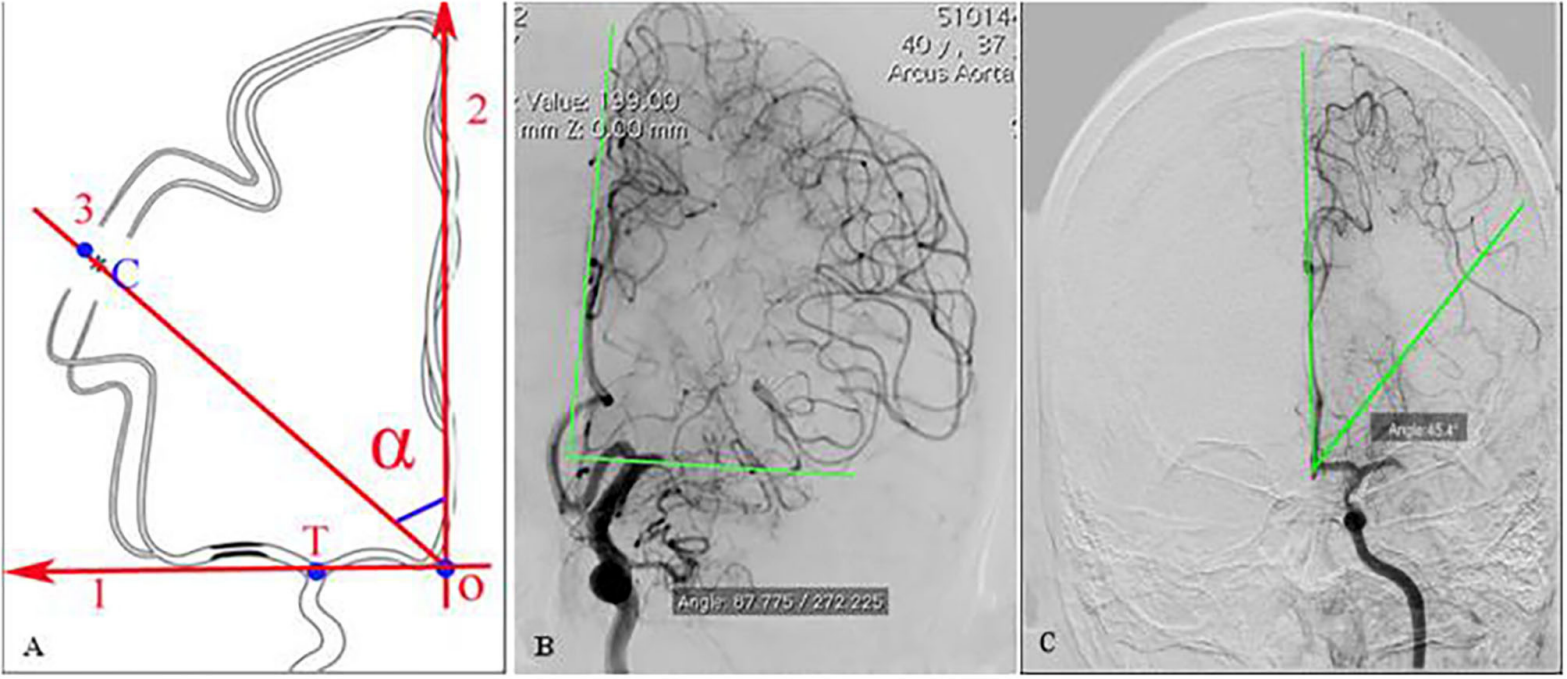
Definition of ABZA on DSA image (with permission) (6). **(A)** determine point T at the intracranial bifurcation of ICA, find the midline 2, that is, the projection of sagittal sinus, the vertex O is obtained from the vertical projection of point T on midline 2, and determine the central point C of the boundary zone of ACA-MCA, with line 2 as the starting edge and OC as the ending edge, and the angle α formed is ABZA. **(B,C)** The central point C was determined by the several farthest points of ACA cortical meningeal arterioles retrograde flows, which was the terminal edge of ABZA in patients with MCAO. **(B)** Demonstrated excellent collaterals with ABZA near to 90°; **(C)** showed a moderate collaterals with ABZA of 45.4°.

The ABZA was measured as previously reported (6). Briefly, the projection of intracranial bifurcation of internal carotid artery on the median line of sagittal suture is defined as the vertex, and the median line of sagittal suture as the starting edge on the DSA image of Tang's position. The angle between the center and the vertices of the ACA-MCA cortical junction (the central point of the junction area was determined according to the points at which the pia mater arterioles of ACA and MCA first met) (Figure 2A). In patients with complete occlusion of MCA, the line between the farthest point of the retrograde collateral circulation and the vertex was selected as the terminal edge (Figures 2B,C). In patients with dysplasia or ACA-A1 deletion (diameter < 0.5 mm) but AcoA was not closed, ABZA was measured from contralateral carotid angiography images. ABZA was 0° when both AcoA and ipsilateral ACA-A1 were not visible (6). The assessment of angiographic images was performed by two experienced neurointerventionalists (Y. CH. and MJ. C) using the image processing software of the GE PACS system to measure ABZA, and any disputes were resolved after consultation. The inter-rater reliability (IRR) was evaluated.

### Classification Method of ABZA-Grading

**Figure 3:**
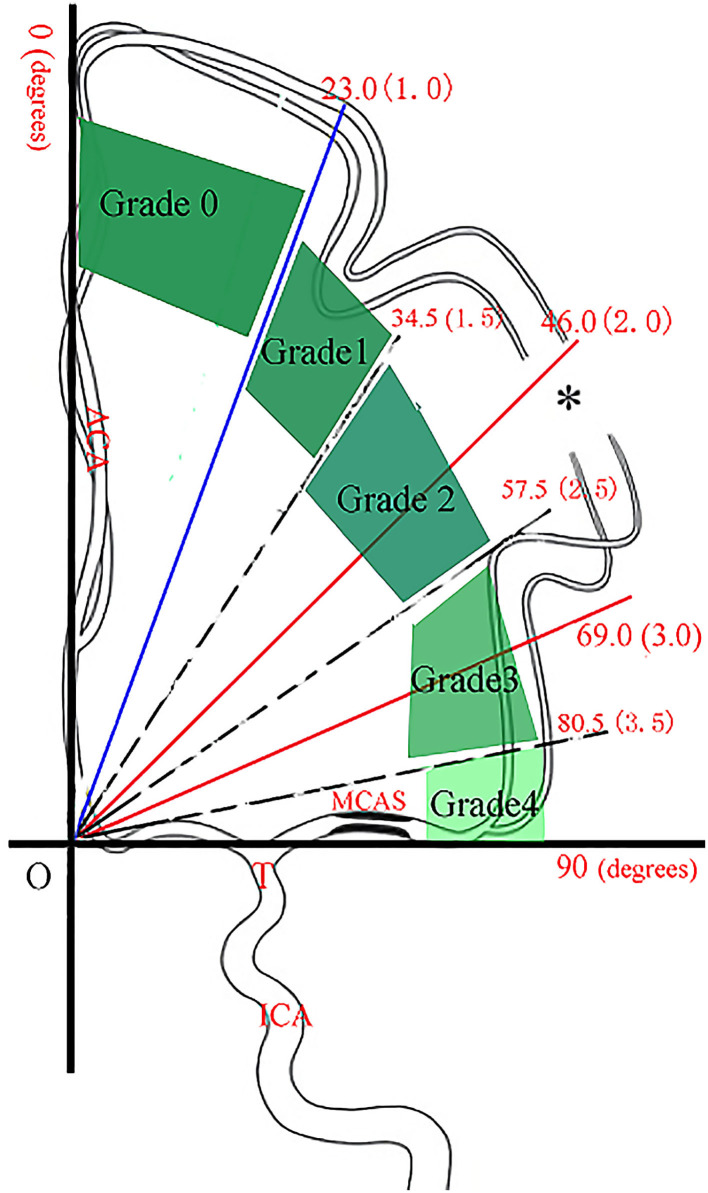
The classification method of ABZA-grading (with permission) (6). On the coordinate graph, point T is the end bifurcation point of internal carotid artery, the vertical line is the middle line of the sagittal sinus, and OT is the vertical line of the middle line. * indicates the central point at which the pia mater arterioles of ACA and MCA first met. The vertical area is divided into four parts from three solid lines, corresponding to ABZA: 23.0°, 46.0°, and 69.0°. In addition, the three dashes correspond to ABZA: 34.5°, 57.5°, and 80.5°. The red number in each bracket indicates the corresponding ABZA-trans (ABZA/23.0°), and when rounded it up, the integer number is the corresponding collateral score (ABZA-grading).

The classification method of ABZA-grading is shown in Figure 3 (6). The ABZA/23.0° (the upper limit of 95% normal reference range of ABZA) was rounded to obtain the corresponding collateral circulation score. ABZA/23.0° is defined as ABZA_trans_: ABZA-grading is 0 if ABZA_trans_ ≤ 1.0; if 1 ≤ ABZA_trans_ < 1.5, ABZA-grading is 1; 1.5 ≤ ABZA_trans_ < 2.5, ABZA-grading is 2; 2.5 ≤ ABZA_trans_ < 3.5, ABZA -grading is 3; 3.5 ≤ ABZA_trans_ < 4.0, corresponding ABZA-grading is 4 (Figure 3).

### Statistical Analysis

IBM SPSS Statistics 22 statistical software was used for statistical analysis. Quantitative data meeting normal distribution were described by mean ± standard deviation ($\bar{x}\;\pm$ s), and quantitative data with non-normal distribution were described by median (M) and inter quartile range (IQR). Univariate analysis was used to compare the baseline data between two groups. Logistic regression analysis was used to analyze the independent predictors of the 90-day clinical outcome. The 90-day functional outcome (mRS) of ABZA-grading group and the ABZA-grading of different TOAST (*Trial of Org 10172 in acute stroke treatment*) types were analyzed by contingency table analysis. Receiver operating characteristic curve (ROC) analysis was used to judge the predictive value of ABZA and the prediction accuracy of each logistic regression analysis model. Previous studies confirmed a linear relationship between ABZA-grading and ASITN/SIR-grading (6). Considering such close interaction, either ASITN/SIR-grading or ABZA-grading was manually eliminated, while the other was retained as collateral circulation score when performing multivariate analysis. In addition, all variables with *P* < 0.05 in univariate analysis were included in logistic regression analysis. *P* < 0.05 was considered statistically significant.

## Results

### Analysis of Baseline Clinical Data

Patients with mRS ≤ 2 or mRS > 2 were stratified into good and poor prognosis groups, respectively (Table 1). A total of 108 patients with acute MCAO treated with EVT were included, including 69 cases (63.9%) in Fujian Provincial Hospital, 39 cases (36.1%) in Quanzhou First Hospital. Among which 63 cases (58.3%) were male. According to TOAST etiological classification and 59 cases (54.6%) were cardiogenic embolism (CE) and 44 cases (40.7%) with LAA. Finally, 37 cases (34.3%) had a good prognosis (mRS ≤ 2) and 71 cases (65.7%) had poor prognosis (mRS > 2). Compared with the poor prognosis group, the good prognosis group were younger [media 61 versus (vs.) 72 years, *P* < 0.001], Furthermore, the proportion of patients with LAA (59.5 vs. 31.0%, *P* = 0.012) was higher in the good prognosis group compared with the poor one. There were no significant differences in baseline ASPECTS, baseline NIHSS, other stroke risk factors (including hypertension, diabetes, hyperlipidemia, coronary atherosclerotic heart disease, stroke history, smoking, and drinking history), hospital, and gender between the two groups.

**Table 1:** The baseline clinical data of patients with and without a favorable outcome.

**Variables**	**mRS > 2**	**mRS ≤ 2**	***P-*value**
	**(*n* = 71)**	**(*n* = 37)**	
**Patients characteristics**, ***n*****(%)**			
Age, y (M, IQR)	72 (64–78)	61 (55–67)	<0.001^**^
Male	38 (53.5)	25 (67.6)	0.160
Hospital, *n* (%)			0.125
Fujian Provincial Hospital	49 (69.0)	20 (54.1)	
Quanzhou First Hospital	22 (31.0)	17 (45.9)	
**Risk factors of stroke**, ***n*****(%)**			
Hypertension	50 (70.4)	24 (64.9)	0.555
Diabetes mellitus	22 (31.0)	7 (18.9)	0.179
Hyperlipidemia	15 (21.1)	11 (29.7)	0.321
CHD	9 (12.7)	4 (10.8)	1.000
History of stroke	15 (21.1)	5 (13.5)	0.334
Smoking	12 (16.9)	9 (24.3)	0.355
Drinking	7 (9.9)	6 (16.2)	0.335
**Clinical characteristics**			
SBP > 140 mmHg, *n* (%)	41 (57.7)	15 (40.5)	0.089
DBP > 90 mmHg, *n* (%)	20 (28.2)	6 (16.2)	0.168
Baseline glucose > 7 mmol/L, *n* (%)	43 (60.6)	17 (45.9)	0.147
Baseline NIHSS (SD)	18.0 ± 5.4	14.1 ± 5.6	0.761
Baseline ASPECTS (M, IQR)	9 (8, 10)	10 (9, 10)	0.053
TOAST types, *n* (%)			0.012^*^
LAA	22 (31.0)	22 (59.5)	
CE	46 (64.8)	13 (35.1)	
Other and unknown reasons	3 (4.2)	2 (5.4)	

*M, median; IQR, inter quartile range; mRS, modified Rankin Scale score; NIHSS, National Institutes of Health Stroke Scale; aspects, Alberta stroke program early CT Score; toast, acute stroke treatment Org 10172 trial standard; AF, atrial fibrillation; CDH, coronary atherosclerotic heart disease; LAA, large atherosclerosis; CE, cardiogenic embolism*.

* *P < 0.05*;

** *P < 0.001*.

### Analysis of Related Variables of Endovascular Therapy

The Inter-rater reliability of ABZA was very good (IRR = 0.78). There was no significant difference in anesthesia mode, requirement for remedial measures (balloon angioplasty or stent implantation), passes of retriever or recanalization, bridging therapy and onset to recanalization time between the good and poor prognosis groups. Compared with poor prognosis group, patients in good prognosis group had higher proportion of good collateral circulation (ABZA-grading > 2) (75.7 vs. 11.3%, *P* < 0.001) and mTICI 2b/3 grade (94.6 vs. 76.1%, *P* = 0.016) (Table 2).

**Table 2:** The EVT related variables of patients with and without a favorable outcome.

**Variables**	**mRS > 2**	**mRS ≤ 2**	***P-*value**
	**(*n* = 71)**	**(*n* = 37)**	
Type of anesthesia, *n* (%)			0.935
Local anesthesia	61 (85.9)	32 (86.5)	
General anesthesia	10 (14.1)	5 (13.5)	
Collateral circulation score, *n* (%)			<0.001^**^
ASITN/SIR-grading			<0.001^**^
ASITN/SIR-grading ≤ 2	65(91.5)	17 (45.9)	
ASITN/SIR-grading > 2	6 (8.5)	20 (54.1)	
ABZA-grading			<0.001^**^
ABZA-grading ≤ 2	63 (88.7)	9 (24.3)	
ABZA-grading = 0	4 (5.6)	0 (0)	
ABZA-grading = 1	28 (39.4)	2 (5.4)	
ABZA-grading = 2	31 (43.7)	7 (18.9)	
ABZA-grading > 2	8 (11.3)	28 (75.7)	
ABZA-grading = 3	6 (8.5)	25 (67.6)	
ABZA-grading = 4	2 (2.8)	3 (8.1)	
Bridging therapy, *n* (%)	29 (40.8)	14 (37.8)	0.762
Remedies, *n* (%)			0.227
Balloon angioplasty	4 (5.6)	4 (10.8)	
Stenting	0 (0)	1 (2.7)	
Onset to puncture, min (SD)	219.9 ± 103.5	221 ± 109.0	0.623
Puncture to recanalization, min (M, IQR)	100 (60–175)	95 (60–120)	0.264
Onset to recanalization, min (SD)	338.3 ± 104.2	323.9 ± 113.2	0.497
mTICI 2b/3, *n* (%)	54 (76.1)	35 (94.6)	0.016^*^
Passes of retriever, *n* (%)			0.157
1	32 (45.1)	20 (54.1)	
2	20 (28.2)	13 (35.1)	
≥3	19 (26.8)	4 (10.8)	
Passes of recanalization, *n* (%)			0.733
1	34 (47.9)	19 (51.4)	
≥2	37 (52.1)	18 (48.6)	

*SD, standard deviation; M, median; IQR, inter quartile range; mRS, modified Rankin Scale score; ABZA-grading, anterior borderzone angle grading; mTICI, modified thrombolytic grade of cerebral infarction*.

* *P < 0.05*;

** *P < 0.001*.

### Logistic Regression Analysis of the Independent Predictors of the 90-Day Clinical Outcome

Univariate analysis of the factors with *P* < 0.05, including age, TOAST types, ASITN/SIR-grading (poor vs. good) and ABZA-grading (poor vs. good), mTICI (poor vs. good) were included in logistic regression analysis by enter method, and two models were established. ROC curve was used to analyze the prediction accuracy of each models. The results are shown in Table 3. Model 1 and model 2 were manually screened out either ASITN/SIR-grading or ABZA-grading, respectively. The AUC of Model 1 was 0.903 (95% CI: 0.844–0.961; *P* < 0.001) and Model 2 was 0.862 (95% CI: 0.794–0.929; *P* < 0.001). The above results suggest that ABZA-grading (OR 18.948; 95% CI: 5.728–62.680; *P* < 0.001), ASITN/SIR-grading (OR 8.529; 95% CI: 2.455–29.634; *P* < 0.001) and age (OR 0.939; 95% CI: 0.892–0.988; *P* = 0.016) are independent predictors of the 90-day clinical outcome, and the model with ABZA-grading had a higher predictive value than ASITN/SIR-grading. In contrast, there were no significant differences in TOAST types, final mTICI 2b/3 between the good and poor prognosis groups.

**Table 3:** Logistic regression analysis of the 90-day-outcome predictors in MCAO patients after EVT.

**Variables**	**Model 1**	**Model 2**
Model description	ASITN/SIR-grading was manually eliminated: enter method	ABZA-grading was manually eliminated: enter method
Age	OR 0.939; 95% CI: 0.892–0.988; *P* = 0.016^*^	OR 0.938; 95% CI: 0.893–0.985; *P* = 0.011^*^
TOAST types (LAA, CE, other)	*P* = 0.314	*P* = 0.215
ASITN/SIR-grading (poor vs. good)	–	OR 8.529; 95% CI: 2.455–29.634; *P* < 0.001^**^
ABZA-grading (poor vs. good)	(OR 18.948; 95% CI: 5.728–62.680; *P* < 0.001**)	–
mTICI (poor vs. good)	OR 5.869; 95% CI: 0.837–41.128; *P* = 0.075	OR 5.712; 95% CI: 1.037–31.463; *P* = 0.045^*^
AUC	0.903 (0.844–0.961)	0.862 (0.794–0.929)

*mRS, modified Rankin Scale score; mTICI, modified thrombolytic grade of cerebral infarction; ABZA-grading, anterior borderzone angle grading; NIHSS, National Institutes of Health Stroke Scale; ASITN/SIR, the American Society of Interventional and Therapeutic Neuroradiology/Society of Interventional Radiology; AUC, The area under ROC curve*;

* *P < 0.05*;

** *P < 0.001*.

### The Predictive Value of ABZA

**Figure 4:**
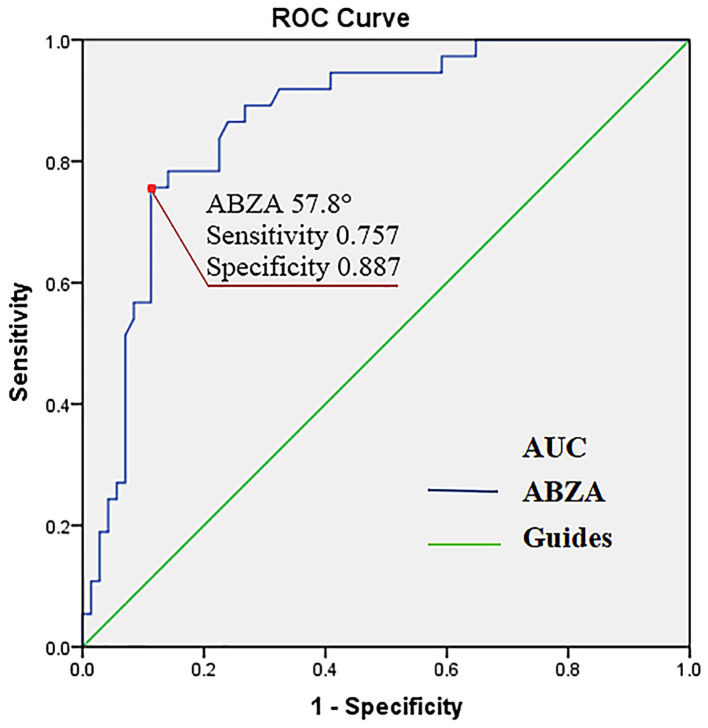
Receiver operating characteristic curve (ROC) of ABZA predicting a favorable 90-day clinical outcome. The area under ROC curve (AUC) of ABZA was 0.868 (95% CI: 0.79–0.938; *P* < 0.001). For an ABZA of >57.8° (corresponding ABZA-grading of >2), the predictive sensitivity and specificity were 75.7 and 88.7%, respectively (the red point). ABZA, anterior borderzone angle.

Sensitivity and specificity of ABZA on disease prognosis was measured by ROC analysis (Figure 4). The area under ROC curve (AUC) of ABZA was 0.868 (95% CI: 0.798–0.938; *P* < 0.001), indicative of ABZA good predictive value for the 90-day prognosis of patients with acute MCAO after EVT. In addition, Youden index of ROC analysis was 0.644, with a best cut-off value of ABZA of 57.8° corresponding to an ABZA-grading of 2. The sensitivity and specificity for predicting favorable prognosis at 90 days were 75.7 and 88.7%, respectively, supporting the hypothesis of ABZA-grading > 2 as an excellent collateral circulation.

### The Analysis of mRS Score According to the ABZA-Grading Subgroups

**Figure 5:**
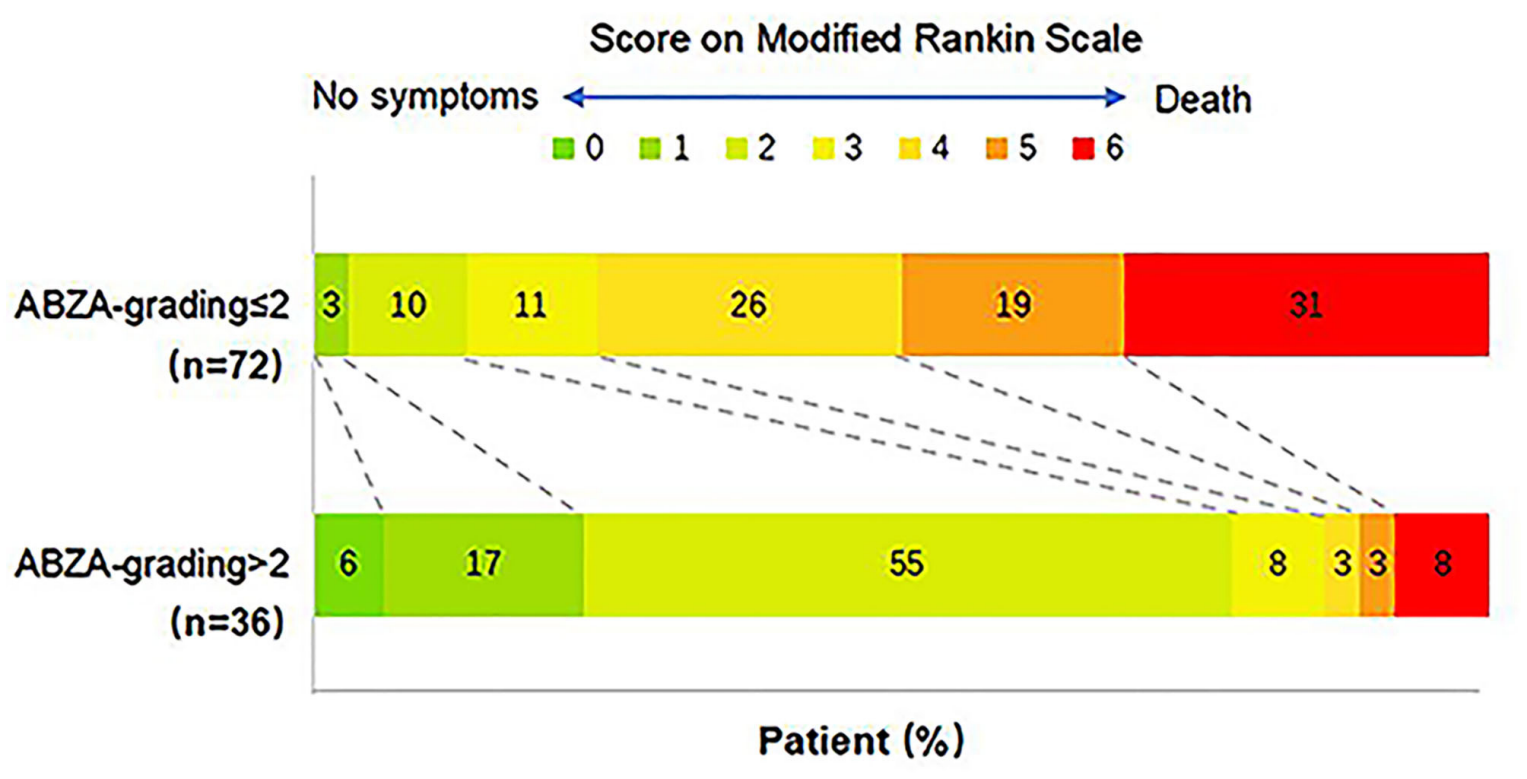
Comparison of the 90-day functional outcomes stratified to ABZA-grading. ABZA-grading ≤ 2 was defined as poor colleral circulation, and ABZA-grading > 2 was defined as excellent collateral circulation. Among them, the cases of mRS ≤ 2 were 9 (13%) and 28 (78%), respectively. Contingency table analysis showed that there were significant differences in mRS score between ABZA-grading subgroups (*P* < 0.001). mRS scores ranged from 0 to 6, with 0 indicating asymptomatic, 1 non-clinically significant disability, 2 mild disability (patients can take care of their own affairs, but cannot carry out all previous activities), 3 moderate disability (patients need some help but can walk independently), 4 severe disability (patients cannot walk independently, cannot take care of their own physical needs), 5 Severe disability (patients need continuous care), 6 death.

The change of mRS score at 90 days in ABZA-grading subgroups are shown in Figure 5. Among ABZA-grading ≤ 2 and ABZA-grading > 2, the cases of mRS ≤ 2 were 9 (13%) and 28 (78%), respectively, the ABZA-grading ≤ 2 group tended to have a higher mRS score. Contingency table analysis was used to analyze the mRS score between the ABZA-grading subgroups (ABZA-grading > 2 vs. ≤ 2). The results showed statistically significant differences in mRS scores between ABZA-grading subgroups (*P* < 0.001).

### ABZA-Grading for Patients With Different TOAST Subtypes

**Figure 6:**
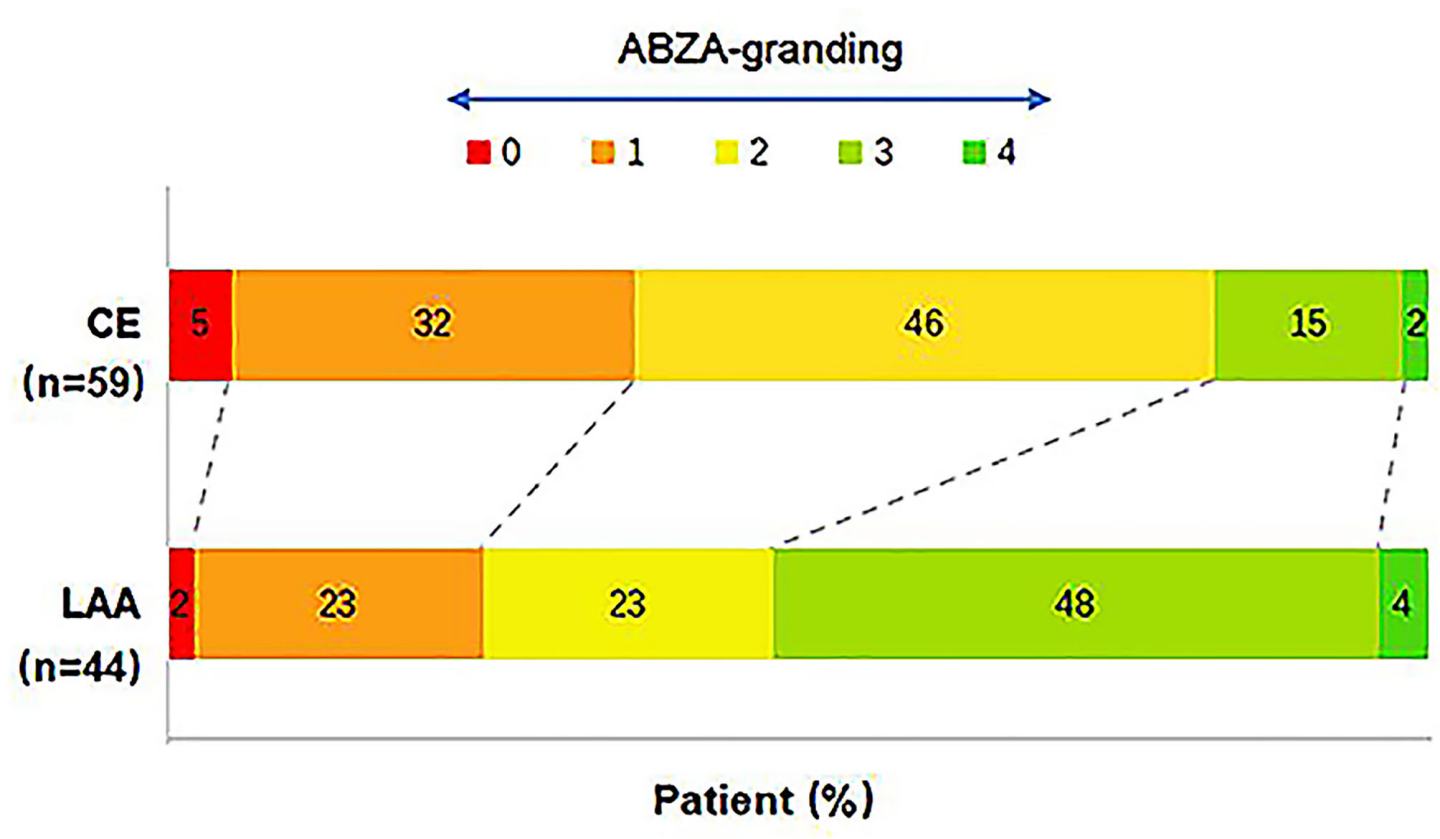
Distribution comparison of ABZA-grading between CE and LAA subgroups. The ABZA-grading scores ranged from 0 to 4. The figure showed that there were 59 cases with CE and 44 cases with LAA. Among them, the cases of ABZA-grading > 2 were 10 (17%) and 23 (52%), respectively. The results of contingency table analysis indicated that the difference of ABZA-grading between the two subtypes of cerebral infarction etiology was statistically significant (*P* = 0.003).

The ABZA-grading distribution between CE and LAA is shown in Figure 6. Contingency table was used to analyze the difference of ABZA-grading between CE and LAA following TOAST classification. The results showed that the difference of ABZA-grading between the two subtypes was statistically significant (*P* = 0.003).

## Discussion

Collateral circulation is an alternate vascularization path that circumvents severely stenosed or occluded vessels and provides natural blood perfusion compensation for ischemic tissues. Collateral circulation is an important determinant of clinical prognosis in patients with AIS (9). Previous studies have shown the importance of collateral circulation. Good collateral circulation plays an important role in prolonging the maintenance time of ischemic penumbra, reducing core infarct volume, improving the recanalization rate, reducing the risk of bleeding transformation after recanalization, increasing the prognosis of good neurological function, and ultimately reducing the risk of death (10). In recent years, many related clinical studies, including MR CLEAN, EACAPE, EXTEND-IA, and SWIFT-PRIME, demonstrated the benefits of EVT in patients with LVO-AIS (11–15). However, many factors limit the effectiveness of EVT and affect the prognosis, such as onset time to treatment, collateral circulation and the equipment and technology of interventional embolectomy.

Nevertheless, animal studies have shown that collateral circulation is transient and gradually attenuates over time (16). Further studies found that elevated intracranial pressure, blood pressure fluctuations, venous steal and collateral thrombosis may attenuate collateral circulation (17, 18). Most collateral circulation can only be maintained for 6 h. For patients with LVO of anterior circulation, regardless of whether there is evaluation of magnetic resonance perfusion imaging, the infarct core volume increases over time (6–24 h). Therefore, the role of collateral circulation has a limited impact on EVT therapeutic window extension. However, patients who can maintain collateral circulation for more than 6 h may benefit from EVT through advanced imaging screening (19). Therefore, the assessment of collateral circulation during angiography can guide the treatment decision of LVO-AIS, screen potential patients and improve the prognosis. Therefore, we aimed to study whether ABZA-grading could act as an independent prognostic factor of a good 90-day clinical outcome in MCAO patients after EVT. Furthermore, we analyzed the predictive value of ABZA-grading for the prognosis of acute MCAO patients after intravascular recanalization.

This study demonstrated that ABZA-grading was an independent predictor of good functional prognosis in LVO-AIS patients after EVT, and that ABZA-grading > 2 has a significant predictive value for a good 90-day clinical outcome.

ASITN/SIR-grading is a classical DSA collateral evaluation method that subjectively reflects the compensation of actual ischemic area (20). In fact, there are few reports on internal consistency of observers. Some researchers also proposed the use of a 4-point system capillary index score (CIS) to evaluate collateral circulation and predict the prognosis of EVT (21). In this grading system, the MCA blood supply area is divided into three equal parts. During the capillary phase of DSA, if there is capillary staining in each part, one point will be recorded. The higher the score, the better the collateral circulation. This method can evaluate the collateral compensation of ACA and posterior cerebral artery (PCA) in patients with MCAO, but is again highly subjective.

From our study, we also found that ABZA-grading may be a slightly stronger predictor than ASITN/SIR-grading. As we know, ABZA is a quantitative index based on the clear boundary anatomical area of ACA and MCA, and in imaging, the terminal edge can be determined according to the initial meeting points of the pial arterioles of ACA and MCA, and the angle between them and the median line of the sagittal sinus is ABZA. In patients with MCAO, the farthest point of retrograde collateral circulation was selected to determine the terminal edge (6). Furthermore, we found that after data transformed (ABZA/95% normal upper limit), the quadrant was divided into four equal parts. According to the degree of retrograde blood flow, the collateral circulation score was very similar to ASITN/SIR-grading. For example, ASITN/SIR-grading 1 was defined as a small amount of collateral blood flow reaching the edge of ischemic area, which corresponded to ABZA slightly > 23° and an ABZA-grading of 1. In complete contrast, ASITN/SIR-grading 4 was defined as complete compensation with retrograde blood flow to M1 bifurcation, corresponded to an ABZA close to 90° and ABZA-grading of 4. Although ABZA-grading and ASITN/SIR-grading resemble each other, ABZA-grading is based on a relatively objective measurement, while the latter one is mainly based on subjective judgment. David Liebeskind et al. had reported an excellent inter-rater reliability (IRR = 0.87) in the International Stroke Conference 2017 (22), and in this study, it was also very good (IRR = 0.78). Therefore, ABZA-grading is more intuitive and objective for clinical application. Nonetheless, repeatability and feasibility across observers needs further clinical research.

However, we did not find the predictive value of time, including time from onset to recanalization, time from onset to puncture, time from puncture to recanalization, as an independent factor for the 90-day clinical outcome. The study of HERMES showed that (23), the favorable 90-day clinical outcome of EVT patients decreased with the extension of the time from onset to puncture. With the progress of stroke, any time delay from onset to treatment will bring significant brain cell damage and death. In addition, the correlation between EVT times, functional prognosis, and clinical outcome of LVO-AIS has been neglected in recent years. Several studies have shown that more than three occurrences of thrombectomy and remedial treatment are associated with delayed reperfusion and increasing the risk of complications (24–27). Meanwhile, repeated thrombectomy can cause vascular damage and damage to the blood-brain barrier, which increases the risk of postoperative intracranial hemorrhage transformation leading to poor prognosis. Recent studies have also found that patients with good collateral circulation have fewer embolectomies to achieve recanalization (28), possibly due to the beneficial impact of cross thrombus pressure gradient, generated by collateral circulation, on thrombus removal. Secondly, collateral circulation can remove micro emboli, thus reducing the distal embolism caused by thrombus escape during thrombectomy. It may be the indirect reason for patients with good collateral circulation to obtain a favorable prognosis. However, this study did not show the relationship between passes of retrieve and a good 90-day clinical outcome. Here, multivariate analysis showed no significant differences in clinical prognostic factors, such as baseline NIHSS, ASPECTS, mTICI between the good and poor prognosis groups. However, this might be a result of the small sample size of this retrospective study. We cannot exclude possible systematic and random errors in the study process, which can cause bias and affect the prognosis analysis results.

In addition, the results of this study show that ABZA-grading is different in different TOAST etiological types (CE and LAA). Compared with CE patients, the proportion of ABZA-grading > 2 in LAA patients is higher, indicating that CE patients may have worse collateral circulation than LAA patients. These results suggest that ABZA-grading may have a significant value to differentiate the etiology of cerebral infarction.

Some animal experimental studies have found that chronic cerebral hypoperfusion can promote the formation and recruitment of new collaterals. Meanwhile the vascular blockage of CE occurs suddenly without chronic cerebral hypoperfusion, by which time the formation of cortical pial collateral circulation is usually too late. In fact this might be the reason for the difference of collateral circulation between the two (29, 30).

In clinical practice, only a few CE patients had good collateral circulation, again suggesting that most LAA patients may have good collateral circulation. Future studies should test this hypothesis by combining clinical data, imaging characteristics, and first pass effect of the microcatheter (31). Similarly, further studies are needed to verify the efficacy of ABZA-grading in predicting the prognosis of EVT and the etiological classification of cerebral infarction in patients with acute MCAO.

Our study has several limitations. First, the ABZA is a planar conception that cannot accurately reflect the real three-dimensional status of the blood supply of the brain. Moreover, it may not reflect the actual status of brain perfusion when some MCA leptomeningeal arteries have disappeared or a large cortical infarction is present (6). Second, all data here presented were extracted from two hospitals, but the neurointerventional physicians in two hospitals received common interventional treatment training. Therefore, EVT adopted a similar scheme, and there was no significant difference in patient characteristics between the two institutions, the reason why these comprehensive data could be used for further statistical analysis. Third, as far as we knew, ABZA is a quantitative index based on the clear boundary anatomical area of ACA and MCA. Therefore, it was not fit to be reliably used in the clinical setting of ICA or MCA-M_2/3_ occlusions. Finally, this study is a retrospective study with a small sample size, which is prone to bias and affects the results. Therefore, we need to test our hypothesis through a multicenter prospective study with a large sample size.

## Conclusion

The ABZA-grading can be an objective and easy assessment of collateral circulation, and is independently associated with short-time clinical outcome in patients with acute MCAO following EVT treatment. Therefore, ABZA-grading and other measurements of collateralisation may guide AIS patient selection for EVT. The ABZA-grading of collateral circulation can be a supplemental metric that helps differentiate stroke by LAA and CE.

## Data Availability Statement

The raw data supporting the conclusions of this article will be made available by the authors, without undue reservation.

## Ethics Statement

The studies involving human participants were reviewed and approved by the Ethics Committee of Fujian Provincial Hospital and Quanzhou First Hospital. The patients/participants provided their written informed consent to participate in this study.

## Author Contributions

YL, YW, and JL conceived, designed, and supervised the study. YC and QH contributed to data analysis, data interpretation, and writing original draft. ZZ, YH, SC, CW, MC, and QC contributed to data collection and image analysis and interpretation. All authors contributed to the article and approved the submitted version.

## Conflict of Interest

The authors declare that the research was conducted in the absence of any commercial or financial relationships that could be construed as a potential conflict of interest.

## Publisher's Note

All claims expressed in this article are solely those of the authors and do not necessarily represent those of their affiliated organizations, or those of the publisher, the editors and the reviewers. Any product that may be evaluated in this article, or claim that may be made by its manufacturer, is not guaranteed or endorsed by the publisher.
