# Supplementary material for: Effects of Anterior Borderzone Angle Grading on Predicting the 90-Day Prognosis After Recanalization of Acute Middle Cerebral Artery Occlusion
Source: Front Neurol. 2021 Aug 26;12:700732. doi: 10.3389/fneur.2021.700732 (PMC8427753; doi:10.3389/fneur.2021.700732)
Supplement: Supplementary file 1 [file Data_Sheet_1.docx]

Supplementary Materials

| **Suppl. Tab1 The baseline clinical data of patients with different ABZA-grading groups** | | | |
| --- | --- | --- | --- |
| Variables | ABZA-  grading ≤2 (n=72) | ABZA-grading >2(n=36) | *P* value |
| Patients characteristics, |  |  |  |
| Age (M, IQR, y) | 71.5 (64.0-77.5) | 61 (55.0-69.0) | <0.001^**^ |
| Male | 40 (55.6) | 23(63.9) | 0.408 |
| Risk factors of stroke (n, %) |  |  |  |
| Hypertension | 51 (70.8) | 23 (63.9) | 0.464 |
| diabetes mellitus | 19 (26.4) | 10(27.8) | 0.878 |
| Hyperlipidemia | 18 (25.0) | 8 (22.2) | 0.750 |
| CHD | 9 (12.5) | 4 (11.1) | 0.833 |
| History of stroke | 14 (19.4) | 6(16.7) | 0.726 |
| Smoking | 13 (18.1) | 8 (22.2) | 0.606 |
| Drinking | 8 (11.1) | 5 (13.9) | 0.917 |
| Clinical characteristics |  |  |  |
| SBP (M, IQR) | 144.0 (132.0-170.5) | 138.0 (122.5-157.5) | 0.046^*^ |
| DBP (M, IQR) | 81.0 (75.0-94.0) | 79.0(70.0-90.0) | 0.112 |
| Baseline glucose (M, IQR) | 7.5 (6.8-10.0) | 7.1 (6.3-8.5) | <0.001^**^ |
| Baseline NIHSS,（SD） | 17.5±5.6 | 14.9±5.7 | 0.899 |
| Baseline ASPECTS (M, IQR) | 9(8, 10) | 10(9, 10) | 0.127 |
| TOAST types (n, %) |  |  | <0.001^**^ |
| LAA | 21 (29.2) | 23 (63.9) |  |
| CE | 49 (68.1) | 10 (27.8) |  |
| Other and unknown reasons | 2 (2.8) | 3 (8.3) |  |

M: median; IQR: interquartile interval; NIHSS: National Institutes of Health Stroke Scale; aspects: Alberta stroke program early CT Score; toast: acute stroke treatment Org 10172 trial standard; AF: atrial fibrillation; CDH: coronary atherosclerotic heart disease; LAA: large atherosclerosis; CE: cardiogenic embolism. ^*^*P*<0.05；^**^*P*<0.001.

| **Suppl. Tab2 The EVT related variables of patients with different ABZA-grading groups** | | | | | |
| --- | --- | --- | --- | --- | --- |
| Variables | ABZA-grading ≤2 (n=72) | ABZA-grading >2(n=36) | | *P* value | |
| Onset to puncture, min (M, IQR) | 206.5 (134.5-290.0) | 240.0 (165.0-305.0) | | | 0.333 |
| Puncture to recanalization, min (M, IQR) | 100 (60.5-170.0) | 96.0 (60.0-132.5) | | | 0.270 |
| Onset to recanalization, min (SD) | 334.4 ± 104.6 | 331.4 ± 113.4 | | | 0.582 |
| mTICI 2b/3 (n, %) | 56 (77.8) | 33 (91.7) | | | 0.074 |
| Passes of retriever (n, %) |  |  | | | 0.155 |
| 1 | 36 (50.0) | 16 (44.4) | | |  |
| 2 | 18 (25.0) | 15 (41.7) | | |  |
| ≥3 | 18 (25.0) | 5 (13.9) | |  | |
| Passes of recanalization (n, %) |  |  | | 0.785 | |
| 1 | 36 (50.0) | | 17 (47.2) |  | |
| ≥2 | 36 (50.0) | | 19 (52.8) |  | |

M: median; SD: standard deviation; m: median; IOR: interquartile interval; ABZA-grading: anterior watershed angle score; mTICI: modified thrombolytic grade of cerebral infarction; ^*^*P* < 0.05; ^**^*P* < 0.001.
